# Acute Poisonings in the Fez-Meknes Region Reported to the Poison Control and Pharmacovigilance Center of Morocco: A 20-Year Retrospective Study

**DOI:** 10.1155/jt/6015251

**Published:** 2025-03-19

**Authors:** Imane Iken, Naima Rhalem, Mohammed Abdessadek, Rachid Hmimou, Abdelmajid Soulaymani, Rachida Soulaymani Bencheikh

**Affiliations:** ^1^Department of Genetics and Biometry, Faculty of Sciences, Ibn Tofail University, Kenitra, Morocco; ^2^Life and Health Sciences Research Laboratory, Faculty of Medicine and Pharmacy, Abdelmalek Essaâdi University, Tangier, Morocco; ^3^Poison Control and Pharmacovigilance Center of Morocco, Rabat, Morocco; ^4^Laboratory of Analysis, Modeling, Engineering, Natural Substances and Environment, Polydisciplinary Faculty of Taroudant, Ibn-Zohr University, Agadir, Morocco

**Keywords:** acute poisoning, database, Morocco, Poison Control Center, prognostic factors, toxicology

## Abstract

Following our research on intoxication cases in Morocco, we conducted an investigation into intoxications in the Fez-Meknes region, one of the 12 regions in Morocco most affected by this problem. The main aim of this study is to report the characteristics of intoxication cases and their management. We deemed it necessary to carry out this study to identify the specificities of the Fez-Meknes region and subsequently propose specific measures to minimize risks. Our registry data were based on intoxication cases reported between 1999 and 2018 by the Poison and Pharmacovigilance Center of Morocco (PPCM). During the study period, 23,550 cases were collected. The median age of the patients was 20 years, with extremes ranging from 1 day to 98 years. The sex ratio (M/F) was 0.67. Gaseous compounds were the most incriminated products, accounting for 36.6% of cases. Among the 18,192 patients with a known outcome, 242 cases resulted in death, representing a case fatality rate of 1.3%. The findings of this work provide the first contributions to current data on the epidemiology of intoxications in the Fez-Meknes region over the last 20 years. These data show that intoxications are frequent and of moderate severity.

## 1. Introduction

Since 1980, the reporting of pesticide poisoning cases has been mandatory under the Ministry of Health Circular No. 19 829DR/BF/MM. This marked the first step in establishing the Toxicovigilance System in Morocco [1]. Over the years, this has led to the creation of a database containing more than 203,000 cases of intoxication, excluding scorpion stings. This database is of considerable value, as it has been the foundation for implementing measures to combat poisonings in Morocco. The objective of our work is to report the clinical profile of intoxication cases recorded in the region of Fez-Meknes, which ranked fourth among the 12 regions of Morocco most affected by this issue in 2019 [2]. The choice of this region was based, firstly, on the presence of the Pharmacotoxicology Laboratory, a collaborating center and an important focal point of the Poison and Pharmacovigilance Center of Morocco (PPCM), at the University Hospital of Fez since 2009. Secondly, the limited number of published studies in this region have primarily focused on a specific type of intoxication (product involved and target population).

## 2. Materials and Methods

### 2.1. Sources of Data

#### 2.1.1. Type and Study Period

This is a retrospective study of poisoning cases reported to the PPCM from January 1, 1999, to December 31, 2018, in the Fez-Meknes region, excluding scorpion sting and envenomation cases, which are monitored separately as part of a national strategy.

**Figure 1:**
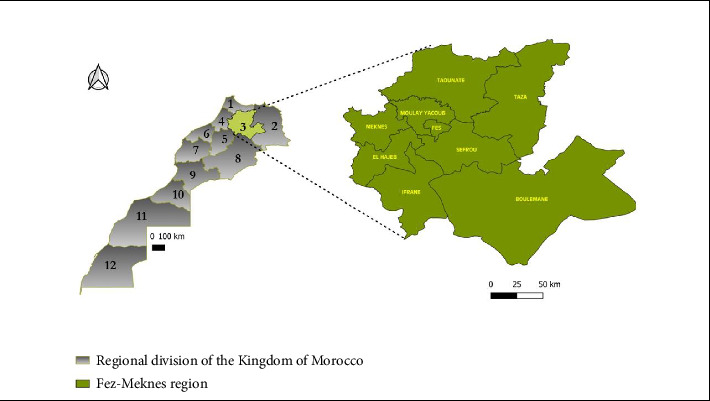
The region of Fez-Meknes, with the different prefectures and provinces [3].

The Fez-Meknes region holds a strategic geographical position and covers an area of 40,075 km^2^, representing 5.7% of the total surface area of the Kingdom of Morocco. According to the general population and housing census, this region had 4,236,892 inhabitants in 2014, accounting for approximately 13% of the country's total population [3]. The population projection for 2020 is 4.405.862 inhabitants, that is, 12.3% of the general population [3] with an observed increase in population between 2014 and 2020. The region includes two prefectures at the administrative level: Fez and Meknes, and seven provinces: Boulemane, El Hajeb, Ifrane, Moulay Yaâcoub, Sefrou, Taounate, and Taza Figure 1.

The data for our study was collected through the following methods.

- Phone calls via the PPCM's phone information service (IT), available 24 h a day, 7 days a week
- Poisoning report forms sent to the PPCM by mail from health professionals at health facilities
- Cases of poisoning gathered through surveys conducted by the PPCM from hospital registers in the region.

#### 2.1.2. Selection of Cases

In our study population, all cases of poisoning reported to the PPCM from the general population or health facilities in the Fez-Meknes region were included. A case of intoxication is defined as follows: “The occurrence of any toxic effect in humans following a single or repeated exposure to a mixture or a natural or synthetic substance, available on the market or in the environment” [4]. All definitions used in the study are based on the official definitions from recognized sources, specifically those provided by the World Health Organization (WHO). Calls to the center for information or educational purposes are not counted as intoxication cases and have therefore been excluded from our research.

### 2.2. Data Collection and Statistical Analysis

All cases received are analyzed on a case-by-case basis. This process ensures the validation of cases according to the definition of poisoning, the completeness of the provided information, and an assessment of causality. It also eliminates duplicate cases [5]. The PPCM follows standardized protocols for analyzing poisoning cases. Data are collected using structured forms through telephone reports, postal submissions, or active surveillance. Missing data are supplemented through follow-ups with reporting entities. Data validation involves cross-referencing information from multiple sources, including healthcare facilities, laboratories, and media monitoring. Regular audits are conducted to address any discrepancies. Analyses are carried out by trained experts, such as toxicologists and epidemiologists, in adherence to national and international standards.

The assessment included characteristics related to the intoxication, the patients, and the products involved. The age classification used was based on the IPCS/WHO guidelines [6]. The severity of intoxication was assessed using the Poisoning Severity Score (PSS) derived from clinical data recorded on the survey forms. The PSS categorizes cases into five severity grades: 0 (*asymptomatic*), 1 (*minor*), 2 (*moderate*), 3 (*severe*), and 4 (*fatal*) [7]. Specific lethality, defined as the percentage of deaths among poisoning cases within a specific category, was calculated to identify substances, circumstances, or populations at the highest risk of mortality. The analysis considered the following parameters, toxic agent types (e.g., plants, pesticides, medications, and food products), demographic characteristics (sex, age groups: infants, children, adults, and elderly), poisoning circumstances (accidental, intentional: suicide and drug abuse), geographical location (urban vs. rural areas), and clinical severity (assessed using the PSS).

For each parameter, deaths were recorded and expressed as a proportion of total cases, generating a lethality profile for each subgroup.

In each poisoning case, the primary substance was identified based on clinical signs, laboratory results, and victim or family reports. Priority was given to the substance most strongly correlated with observed symptoms, ensuring consistent and accurate analysis.

### 2.3. Data Analysis

The collected data were analyzed using a descriptive approach with the Statistical Package for the Social Sciences (SPSS) (Version 21). Quantitative variables were expressed as the mean, standard deviation (Mean ± SD), median, and range, while qualitative variables were presented as numbers and percentages. A bivariate analysis was conducted to assess statistical relationships between variables using the chi-squared (*χ*^2^) test. In addition, the relative risk (RR) was calculated to compare the risks associated with different variables.

## 3. Results

### 3.1. Characteristics of the Study Population

**Figure 2:**
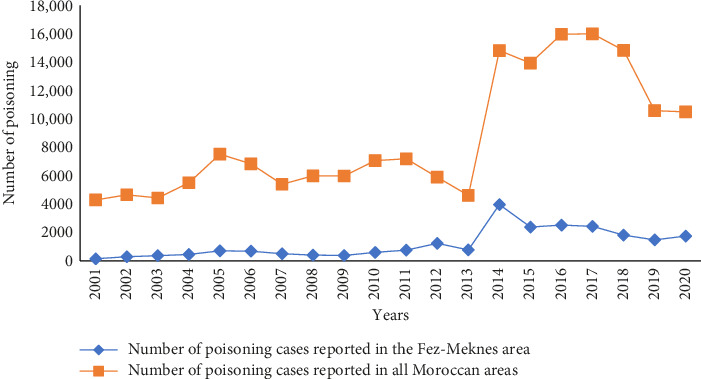
Annual distribution of reported intoxication cases in the area under study and at national level, PPCM, 1999–2018 number of poisoning.

During the study period, the PPCM recorded 23,550 cases of intoxication, excluding envenomation's and scorpion stings. The Fez-Meknes region accounted for 13% of all cases collected by the PPCM. These cases were reported in 62.6% of instances by mail, 28.8% by phone, and 8.6% were gathered from hospital registers. The average incidence of intoxication in the region over the last five years (2014–2018) was 46.5 per 100,000 inhabitants. The increase in cases in this region followed the same pattern as at the national level (Figure 2). The geographical distribution of cases showed that all provinces in the region were affected, with the highest number of cases reported from the prefecture of Fez (27%), followed by the prefecture of Meknes (26%) and the province of Ifrane (22%) (Table 1). Cases were reported seasonally, with 28% occurring in winter, 27% in spring, 24% in autumn, and 21% in summer. The majority of cases (83%) occurred in accidental situations, while 14% were intentional, and 3% had unknown causes (Table 2). The primary routes of exposure were oral (70.2%), inhalation (19.7%), and skin (6.8%). The median age of patients was 20 years, ranging from 1 day to 98 years. The male-to-female ratio was 0.67. Most poisonings occurred in adults (48%), with 74% of cases reported in urban areas compared to 26% in rural areas. Poisonings predominantly occurred at home (77%) and less frequently in public places (12%) (Table 1). The most intoxication was isolated (76%), while they were collective in 24% of cases.

In terms of symptoms, 35% of cases were asymptomatic, while 65% presented with symptoms. The most common clinical manifestations were gastrointestinal disorders (59%) and neuropsychiatric disorders (21%) (Table 1). The severity of intoxication was predominantly moderate (Grade 2) in 56% of cases.

Therapeutic management was initiated for 20,557 individuals, of whom 21% were treated before contacting the PPCM, while 79% were treated following PPCM recommendations (Table 3). Symptomatic treatment was advised in 74% of cases, addressing vital, respiratory, and circulatory failures, as well as convulsions. Antidote treatment was recommended in 70% of cases, and medical monitoring was advised in 97%.

The outcome was favorable in 80% of cases, with 242 deaths recorded, resulting in a case fatality rate of 1%. However, the outcome remained unknown in 19% of cases. Lethality was higher in males (1.6%) compared to females (1.1%) and was significantly higher in neonates (8.8%) compared to adults (1.6%). Geographically, rural areas had a higher lethality rate (2.6%), with the province of Taounate recording the highest lethality at 3%.

### 3.2. Products Involved

Table 1 presents the number of reports and specific lethality rates based on the families of products involved. Poisoning by gaseous products, primarily carbon monoxide, accounted for the highest number of cases in the region, representing 36.5% of the total. Drug poisoning ranked second, followed by food poisoning, with 18.9% and 16.3% of cases, respectively.

However, plant poisoning exhibited the highest lethality rate at 11.7%, followed by drug poisoning at 5.3% and pesticide poisoning at 4.7%. Among plant poisoning cases, *Atractylis gummifera* was responsible for 22.5% of incidents, with a significant contribution to mortality, resulting in 17 deaths (7% of all poisoning deaths in the region). Factors of severity in the evolution of intoxicated persons.

Table 4 highlights the factors influencing the severity and progression to death in cases of intoxication. Neonates showed a significantly higher risk of progressing to death, with a RR of 6.55 compared to other age groups, and a 95% confidence interval (CI) of 2.81–15.31. Rural patients had a 2.57 times greater risk of fatal outcomes compared to urban patients (RR = 2.57; 95% CI: 1.95–3.40).

The findings also revealed that patients who developed cardiac damage had a significantly higher risk of death, with a RR of 12.8 (CI 95%: 7.62–21.54). In addition, patients with herbal intoxication faced a markedly increased risk of mortality, with a RR of 11.20 compared to other toxicants, and a confidence interval of 7.56–16.59.

## 4. Discussion

Over the last decades, acute poisoning has emerged as a major global public health problem. According to the WHO, unintentional poisoning was responsible for an estimated 106,683 deaths and the loss of 6.3 million healthy life years (measured as disability-adjusted life years) in 2016 [8]. The exact incidence of poisoning in Morocco remains unknown due to the lack of comprehensive registry data. In many countries, poisoning is one of the primary reasons for emergency hospital visits, representing a time-sensitive condition that requires specialist intervention for accurate diagnosis and effective treatment.

Based on our findings, the Fez-Meknes region accounted for 13% of the cases reported to the PPCM, excluding envenomation and scorpion stings. Due to the absence of prior studies describing the epidemiological profile of poisoning in the Fez-Meknes region under the current regional division, a comparative analysis of the data is limited. However, according to data collected by the PPCM, 78,374 cases of poisoning were reported between 1980 and 2007 (excluding scorpion stings and envenomation) across Morocco's 16 regions under the former regional division. Hence, we find that the regions of Meknes-Tafilalt, Fez-Boulemane, and Taza-Hoceima-Taounate represented 7.8%, 2.8%, and 2.3% of cases, respectively, amounting to a total of 12.9% of all poisoning cases collected during the same period [9]. These previous findings of intoxication cases closely align with our results (13%). Overall, the data demonstrate a notable and consistent increase in notifications, with a minimum of 143 notifications in 1999 and a peak of 3966 notifications in 2012. The observed rise in reports in the Fez-Meknes region mirrors the trend of progression at the national level.

This significant increase in declarations can be attributed to the implementation of various health vigilance measures by the PPCM. The first axis of this strategy focuses on promoting notifications at both national and regional scales, as well as active case collection through comprehensive surveys. The establishment of the Pharmacotoxicology Laboratory at the Hassan II University Hospital of Fez in 2009 and the introduction of toxicology as a medical discipline at the Faculty of Medicine and Pharmacy of Fez in 2014 have undoubtedly facilitated closer sensitization of medical staff, resulting in an increased number of notifications in the Fez-Meknes region. Our study further revealed that intoxication is more prevalent among young adult females, consistent with findings in the national context [9]. These results are also consistent with data from upper-middle, lower-middle, and low-income countries [10, 11]. However, these results are not consistent with findings from other countries, particularly high-income and upper–middle-income countries [12, 13], where the most affected age group is children aged 0–4 years, accounting for 48.5% of cases in the United States of America and 37% in Italy [14, 15]. In our series, the circumstances of intoxication were predominantly unintentional in 83% of cases, aligning well with previously published data [12, 16–18]. The most frequently involved class of toxicant families in our study was gaseous products, followed by drugs and food. The high frequency of poisoning by gaseous products can be attributed to the region's cold winter climate and the associated use of heating equipment. According to the previously published data [19], in high-income countries, drug intoxications are the leading cause of poisoning. Morocco, however, due to its geographical location at the crossroads of Europe and Africa, exhibits a distinct pattern of agents involved in intoxications. In our case series, plant poisoning showed the highest specific lethality rate (11.7%), followed by pesticide poisoning (5.4%). This can be attributed to the prevalent use of traditional pharmacopoeia in the region, which is influenced by factors such as accessibility, cultural practices, and the abundance of medicinal plants in the Fez-Meknes region. Notably, *A. gummifera* is highly accessible to children, contributing significantly to the high lethality rate within this demographic [20]. In the region covered by our study, the severity of intoxication was moderate in 56% of cases. This finding contrasts with the national profile, where 42% of cases were reported as asymptomatic [21]. Comparing our findings with those of other studies from French poison centers, we observed that the severity of intoxications reported to the CAP in Angers ranged between 85.6% and 93.6% for cases with low or no severity, depending on the year. Similarly, at the CAP in Bordeaux, 84.2% of cases in 2015 fell into the low or no severity category [22]. In contrast, our series highlighted a poorer prognosis primarily associated with plant intoxications, particularly *A. gummifera*, which is consistent with the previously published data [23, 24].

## 5. Conclusion

The primary goal of the current study was to document the clinical profile of intoxication cases in the Fez-Meknes region. Overall, the findings represent the first significant contribution to the epidemiological data on poisoning in this region over the past 20 years. Furthermore, the study revealed that intoxications in the Fez-Meknes region are both frequent and generally of moderate severity. The neonate, rural environment, type of toxin (plants, pesticides, and drugs), and cardiac damage are factors associated with the evolution toward death. This study also highlighted that intoxications are frequent and often serious, necessitating emergency medical care guided by reliable toxicological knowledge. Prevention is crucial and can be achieved through public education, particularly for parents, about the importance of storing hazardous products out of children's reach, adhering to usage instructions, and avoiding the transfer of substances into nonoriginal containers.

Prevention efforts should also involve collaboration with health professionals, encouraging them to consult the PPCM for guidance on the severity of exposed cases. In addition, cooperation with industrial stakeholders is essential, including enforcing regulations on product labeling and packaging, mandating safety caps, banning certain hazardous substances, monitoring contraband products, and ensuring the provision of safety data sheets to the PPCM.

Authorities must also conduct the necessary inspections to ban the sale of dangerous plants permanently and regulate the herbalist profession. These preventive measures could significantly reduce the severity of poisoning cases and the associated healthcare costs.

Strengthening the training of health professionals and raising awareness among the general population in the region are also key strategies to minimize risks. These represent significant challenges but are essential steps to improve the management and outcomes of poisoning cases in the Fez-Meknes region.

## Figures and Tables

**Table 1:** Distribution of poisoning cases according to the characteristics of the poisoning and the intoxicated patient and the toxic agents.

	Overall	Favorable outcome	Death	Frequency of lethal cases
*Prefecture/Province*				
Meknes	3247	3195	52	2.0%
Fez	5380	5282	98	2.0%
Ifrane	4825	4816	9	0.0%
Taza	2679	2634	45	2.0%
Sefrou	900	892	8	1.0%
Taounate	479	463	16	3.0%
Boulemane	362	353	9	2.0%
El Hajeb	245	240	5	2.0%
Moulay Yaâcoub	73	73	0	0.0%

*Area*				
Urban	13,364	13,225	139	1.0%
Rural	3075	2994	81	2.6%

*Sex*				
Female	10,306	10,184	122	1.2%
Male	6963	6852	111	1.6%

*Circumstance*				
Accidental	15,280	15,126	154	1.0%
Intentional	2341	2267	74	3.0%
Unknown	447	436	11	2.0%

*Age range*				
Adult (20–74 years)	7812	7684	128	1.6%
Child (5–14 years)	2538	2505	33	1.3%
Adolescent (15–19 years)	2208	2173	35	1.6%
Toddler (1–4 years)	2037	2020	17	0.8%
Infant (4 week–12 month)	147	145	2	1.4%
Elderly (> 75 years)	103	102	1	0.9%
Neonate (< 4 week)	68	62	6	8.8%
Age not specified	1503	1498	5	0.3%

*Presentation*				
Asymptomatic	3827	3775	52	1.4
Symptomatic	9349	9233	116	1.0
- Digestive disorders	5489	5443	46	0.8
- Neuropsychiatric disorders	2008	1977	31	1.5
- General system disorders	744	734	10	1.3
- Respiratory disorders	457	454	3	0.7
- Cardiac disorders	158	139	19	12.0
- Mucocutaneous disorders	449	443	6	1.3
- Metabolic disorders	36	35	1	2.8
- Others	8	8	0	0.0

*Agents*				
Gas products	6948	6888	60	0.8
Drugs	3061	3041	20	0.6
Food	2970	2955	15	0.5
Pesticides and agricultural products	1543	1470	73	4.7
Venomous animals	1217	1207	10	0.8
Household products	654	651	3	0.5
Industrial products	549	545	4	0.7
Plants	273	241	32	11.7
Narcotics	245	232	13	5.3
Cosmetics	85	84	1	1.2
Others	32	32	0	0.0
Unknown product	615	604	11	1.8

**Table 2:** Distribution of poisoning reports according to the circumstances, PPCM, Fez-Meknes region (1999–2018).

Circumstance	Under circumstance	Head count	Percentage
Accidental	Classic	17,380	74.8
	Food	889	3.8
	Medication error	607	2.6
	Adverse effect	217	1.0
	Professional	146	1.0
	Others	15	0.0

Intentional	Suicide and suicide attempts	3015	13.0
	Drug addiction	220	1.0
	Abortion	18	0.0
	Criminal	39	0.0
	Others	23	0.0

Unknown	Unknown	657	2.8

*N*		23,226	100

**Table 3:** Distribution management of poisoned patients N.

Type of care	Before calling the PPCM		Referred by the PPCM	
	Number (N)	Percent (%)	Number (N)	Percent (%)
Therapeutic abstention	11	1	1228	99
Evacuator treatment	870	30	2035	70
External decontamination	22	11	183	89
Induced nausea	281	37	470	63
Gastric lavage	567	29	1382	71
Symptomatic treatment	3086	26	8690	74
Antidote	270	30	619	70
Medical monitoring	98	3	3637	97
Total	4335	21	16,209	79

**Table 4:** Severity factors related to the patient's evolution.

Variables	Studied variables	Favorable	Death	RR⁣^∗^	IC⁣^∗∗^ 95%
Age groups	Neonate	62	6	6.5	2.8–15.3
	Other age groups	14,629	216		

Environment	Rural	2994	81	2.6	1.9–3.4
	Urban	13,225	139		

Toxic products	Narcotics	232	13	4.3	2.4–7.7
	Other toxic products	17,718	229		
	Pesticides	1470	70	4.8	3.7–6.4
	Other toxic products	16,480	169		
	Plants	241	32	11.2	7.6–16.6
	Other toxic products	17,709	210		

Type of organ involvement	Heart disease	139	19	12.8	7.6–21.5
	Other types of organ involvement	9094	97		

Circumstance	Intentional	2267	74	3.0	2.3–4.0
	Other circumstances	15,683	168		
	Unknown	557	14	1.9	1.1–3.3
	Other circumstances	17,393	228		

⁣^∗^RR: relative risk.

⁣^∗∗^IC 95%: 95% confidence interval.

## Data Availability

The data used to support the findings of this study are available from the corresponding author upon reasonable request.
